# The enigma of infra-slow fluctuations in the human EEG

**DOI:** 10.3389/fnhum.2022.928410

**Published:** 2022-08-02

**Authors:** Juri D. Kropotov

**Affiliations:** N.P. Bechtereva Institute of the Human Brain of Russian Academy of Sciences, Saint Petersburg, Russia

**Keywords:** infra-slow fluctuations (ISFs), full band EEG, BOLD signal of functional MRI, rhythmic and arhythmic EEG-ISF, BOLD signal

## Abstract

Spontaneous Infra-Slow Fluctuations (ISFs) of the human EEG (EEG-ISFs) were discovered 60 years ago when appropriate amplifiers for their recordings were designed. To avoid skin-related artifacts the recording of EEG-ISFs required puncturing the skin under the electrode. In the beginning of the 21st century the interest in EEG-ISFs was renewed with the appearance of commercially available DC-coupled amplified and by observation of ISFs of the blood oxygen level–dependent (BOLD) functional magnetic resonance imaging signal at a similar frequency. The independent components of irregular EEG-ISFs were shown to correlate with BOLD signals which in turn were driven by changes in arousal level measured by galvanic skin response (GSR), pupil size and HRV. There is no consensus regarding the temporal organization of EEG-ISFs: some studies emphasize the absence of peaks on EEG-ISFs spectra, some studies report prominent oscillations with frequency around 0.1 or 0.02 Hz, while some emphasize multiple discrete infraslow oscillations. No studies used parameters of EEG-ISFs as neuromarkers to discriminate psychiatric patients from healthy controls. Finally, a set of working hypotheses is suggested that must be tested in future research to solve the enigma of EEG-ISFs.

## Introduction

Spontaneous Infra-Slow Fluctuations (ISFs) of the human EEG (EEG-ISFs) were discovered in the middle of the 20th century, when appropriate devices (such as chopper amplifiers) for their recordings were designed. The pioneers in this field were Nina A. Aladjalova from the Institute of Psychology in Moscow and Valentina A. Iliiukhina from the Institute of experimental medicine in Saint-Petersburg (Aladjalova, 1957; Iliukhina, 2013). The authors found two types of infra-slow oscillations in the human brain with periods of 10 and 30–90 s, respectively. In studies of Valentin B. Gretchin and Juri D. Kropotov, infra-slow oscillations were found in local oxygen concentrations measured by a polarographic method in the basal ganglia and thalamus in patients to whom gold electrodes were stereotactically implanted for diagnosis and therapy (Kropotov and Grechin, 1975). Different patterns of performance in functional tasks were shown for the rising (increasing) and falling (decreasing) phases of these oscillations (Kropotov, 1979).

In Austria, research on EEG-ISFs of the human brain was carried out by Herbert Bauer and Gezilher Guttmann from Vienna University (Guttmann and Bauer, 1984). In Germany, Brigitte Rockstroh and Niels Birbaumer made a considerable contribution by studying contingent negative variation (CNV) waves in healthy subjects and psychiatric patients and by introducing slow-cortical potentials-based neurofeedback for treating some psychiatric conditions (Lutzenberger et al., 1980; Birbaumer et al., 1990; Rockstroh et al., 1993).

Research in these early years was mostly associated with empirical questions of how EEG-ISFs behave in cued tasks, of how they modulate the quality of brain functioning during continuous performance tasks. In particular, it was demonstrated that slow cortical potentials and psychophysical performance had a U-shaped correlation so that the best performance was associated with small negative shifts and the worst with positive or large negative shifts (Lutzenberger et al., 1979).

At the beginning of 21st century, the interest in EEG-ISFs was renewed by the appearance of commercially available DC-coupled amplified and by observation of ISF in the blood oxygen level–dependent (BOLD) functional magnetic resonance imaging (fMRI) signal (Fox and Raichle, 2007; Power et al., 2014). These fluctuations correlated within brain regions, thus reflecting specific functional connectivity (FC) patterns such as the default mode network and several task-positive networks (Yeo et al., 2011; Margulies et al., 2016). Approximately at this time neurofeedback protocols based on parameters of spontaneous EEG-ISFs have been suggested and applied in clinical practice (Othmer et al., 2013).

Recently, several reviews on neuroscience of EEG-ISFs have been published (Palva and Palva, 2012, 2018; Watson, 2018). In this paper, a brief review of the available data is followed by formulation of a set of working hypotheses needed for establishing a theoretical background for EEG-ISFs.

## Hypothesis and theory

### Requirements for the recording procedure

For reliable recording of EEG-ISFs especially over longer intervals, a DC-coupled amplifier with high input impedance and wide dynamic range is required. Here DC stands for Direct Current in contrast to Alternative Current (AC). Until recently most of the EEG amplifiers were AC-coupled and were incapable of accurately recording EEG-ISFs (Bauer et al., 1989). About 20 years ago, several clinically suitable DC-coupled amplifiers became available. Such amplifiers show sufficiently high input impedance and DC stability as well as a wide dynamic range (±100 mV or higher). In addition, detection of conventional EEG frequencies requires a high sampling rate (250 Hz or higher) of the acquisition software. Those kinds of devices have been used for recording a genuine full-band EEG (FbEEG) (Vanhatalo et al., 2005).

The electrodes for EEG-ISFs recording must be non-polarizable since all polarizable electrode materials (such as gold, tin, platinum, or steel) are coupled in a mainly capacitive manner because of the double electrical layer between the metal and electroconductive gel (Tallgren et al., 2005). Among the currently available electrodes, only those based on Ag/AgCl are adequate. The reusable sintered contact elements demonstrate the best results (Tallgren et al., 2005). These electrodes are absorbent and should be cleaned immediately after use with distilled water. It is also recommended to soak them in a saline solution for up to an hour before use. Chloride is required in the gel for stable operation (Tallgren et al., 2005). A DC-stable skin–gel contact is required for the prevention of gel drying.

### Susceptibility to artifacts

EEG-ISFs are susceptible to contributions from numerous sources, including eye and head movement artifacts, and variations in skin properties. Eyeblink and lateral eye movement artifacts are usually corrected using independent component analysis (ICA) by zeroing the activation curves of individual independent components associated with the corresponding eye movement artifacts (Jung et al., 2000).

The integrity of the skin-electrode interface is crucial to minimizing electrode movement and galvanic skin response (GSR) artifacts. The best way to avoid electrode movement artifacts is to fix the electrode with collodion (Vanhatalo et al., 2005). The best way to avoid GSR artifact is to short circuit the skin by puncturing the skin under the electrode (Picton and Hillyard, 1972).

### Infraslow fluctuations in neuronal recordings

**FIGURE 1:**
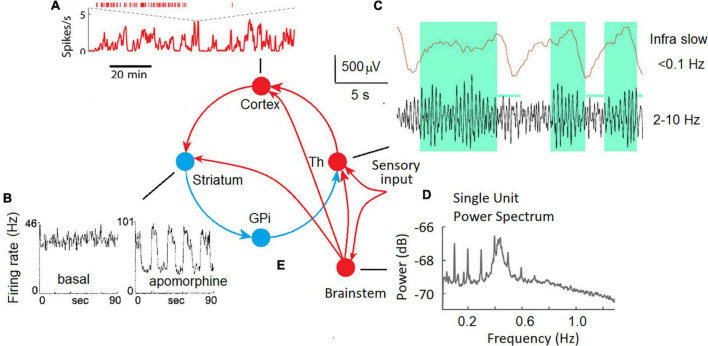
ISF in neuronal activity of different brain areas in animal experiments. **(A)** Fast and slow timescale dynamics of a cortical neuron in the mouse medial prefrontal cortex (Okun et al., 2019). **(B)** The spike trains for a representative neuron in the globus pallidus recorded in an awake rat in the background (basal), and in response to a dopamine agonist (apomorphine) (Ruskin et al., 1999). **(C)** Local field potentials in the lateral geniculate body of the thalamus of a freely moving cat; upper trace – ISF, bottom trace – conventional EEG filtered at 2–10 Hz, note that periods of increased alpha activity are associated with positive-going deflections in infra slow fluctuations (Hughes et al., 2011). **(D)** The plot shows the power spectrum averaged across all single units. Two oscillatory regimes at 0.1 Hz and between 0.4 and 0.5 Hz were observed in single-unit spike trains (Totah et al., 2018). **(E)** Cortical-basal ganglia-thalamocortical loop with excitatory (red) and inhibitory (blue) pathways (Alexander et al., 1990) and the thalamic sensory input.

In animal experiments, ISFs were observed at the micro-level of single neurons in different parts of the brain, including the cortex, basal ganglia, and thalamus (Figure 1).

Two distinct time scales (fast and infra-slow) were discovered at a single neuron level in the mouse medial prefrontal cortex (Figure 1A; Okun et al., 2019). The authors speculate that these two scales in the dynamics of neuronal spiking are regulated by distinct mechanisms: local synaptic factors at the fast time scale and global factors such as slow shifts of arousal level on the slow time scale.

In extracellular single-unit recordings in awake, immobilized rats, many tonically active neurons in the globus pallidus and substantia nigra showed ISFs in firing rate (Ruskin et al., 1999). The dopamine agonist (apomorphine) profoundly affected ISFs by increasing the frequency and regularity of the oscillations. Periodic oscillations in the basal ganglia output nuclei strongly affect the firing patterns of target neurons in frontal-parietal cortical areas *via* the cortical-basal ganglia-thalamocortical loop (Figure 1E) and are likely coordinate motor and cognitive functions (Figure 1B).

Infra-Slow Fluctuations were consistently observed in the thalamus both in slices of isolated thalamic relay nuclei and *in vivo* (Figure 1C; Hughes et al., 2011). A potential role for astrocytes in the genesis of those ISFs as well as a modulatory relationship between them and ongoing alpha oscillations recorded from the scalp was emphasized (Hughes et al., 2011). Infra-slow modulation of gamma oscillations was found in the mouse visual system (Orlowska-Feuer et al., 2021).

A possible role of neuromodulatory (noradrenergic, serotonergic, dopaminergic, and cholinergic) pathways from the brain stem and basal forebrain in the genesis of synchronous ISFs of the cortical neurons must be taken into account. For example, infra-slow (0.01–1 Hz) fluctuations of the spike rate of neurons in the rat locus coeruleus were observed (Totah et al., 2018) suggesting a partly differentiated and target-specific noradrenergic ascending pathways to the cortical areas (Figure 1D). Emergence of infraslow fluctuations in a neuronal model with dopaminergic plasticity was demonstrated as a support of experimental observation of ISF in the globus pallidus (Ruskin et al., 1999) induced by the dopamine agonist apomorphine. Taken into account interaction between the neuromodulatory systems and their effect on slow process such as fluctuations in arousal, effort, sustained attention, cognitive demands, and emotional states (Avery and Krichmar, 2017) participation of these systems in the generation of at least rhythmic ILFs can’t be excluded. In support of this view, the neuronal population rate at infraslow frequencies was highly correlated with arousal level as reflected by the pupil area (Okun et al., 2019).

Although most of the studies of impulse activity of neurons were made in animals, a few studies on patients with electrodes implanted for diagnostic procedures were also available. In the 1970s in our laboratory, we had a unique opportunity of simultaneous recording of multi-unit activity and ISFs in local pO_2_ from gold electrodes implanted for diagnosis and treatment of patients with Parkinson’s disease (Gretchin and Kropotov, 1979). The local pO_2_ was recorded by means of a polarographic method, the electrodes were implanted in the basal ganglia and thalamus. The pattern of fluctuations of multi-unit current rate was different from that of pO_2_: changes in impulse activity looked like shifts between the steady states evoked by either internal or external stimulation. The pO_2_ fluctuations looked like periodic oscillations with frequencies 0.02–0.2 Hz that varied spatially so that the two electrodes with 3 mm apart might show asynchronous oscillations with different frequencies. The frequency of oscillation might change in time sporadically during resting state or in response to the task load.

### Rhythmic vs. arrhythmic EEG-ISFs

In EEG research, the resting state oscillations (rhythms) are usually emphasized. On EEG spectra the oscillations are expressed in a form of local peaks. There are several types of rhythms in the cortex with different underlying mechanisms and different roles in brain dysfunction (for a recent review see Kropotov, 2016).

Irregular, arrhythmic patterns can be also seen in human EEG. The arrhythmic patterns in the EEG are less studied. What we know is that EEG power spectra of arrhythmic EEG obey a simple rule: the power dramatically decreases with frequency. Mathematically the arrhythmic component of EEG spectra can be well approximated by a power-law function: *P*(*f*) = 1/*f^b^*, where *P* is EEG power, *f* is frequency, and *b* is a parameter (typically between 0 and 3) named the “power-law exponent.” In the double-logarithmic coordinates the $\frac{1}{f^{b}}$ function appears as a straight line (He et al., 2010; Palva and Palva, 2018). This law seems to reflect critical-like, scale-free dynamics of EEG fluctuations at various timescales, ranging from seconds to hundreds of seconds (He et al., 2010; Palva and Palva, 2018).

There is no consensus regarding rhythmic EEG-ISFs. Some studies emphasize the absence of peaks on EEG-ISF spectra (Sihn and Kim, 2022a), some studies report prominent oscillations with frequency around 0.1 Hz (Girton et al., 1973; Trimmel et al., 1990; Nikulin et al., 2014) or 0.02 Hz (Watson, 2018), while some studies emphasize multiple discrete infraslow oscillations (Palva and Palva, 2012).

**FIGURE 2:**
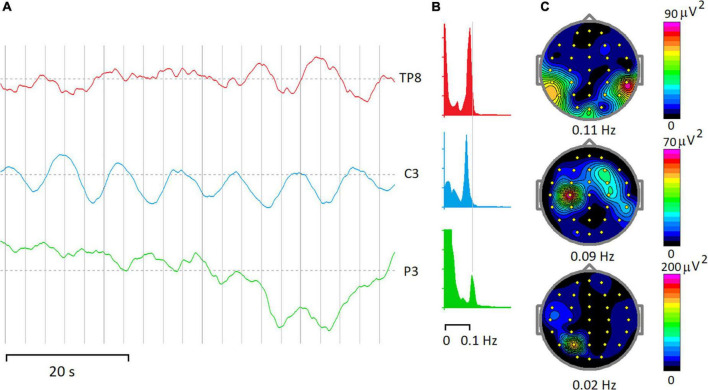
Rhythmic and arrhythmic EEG-ISF. **(A)** Fragments of EEG-ISF in a subject during a continuous performing task. **(B)** Power spectra of 20-min of the EEG record. **(C)** Maps of EEG spectra at the frequencies depicted below. Note that peaks at 0.09–0.11 Hz dominate spectra at TP8 and C3 electrodes, while a power spectrum below 0.1 Hz shows the highest magnitude for the P3 electrode.

Below, the physiological bases of rhythmic and arhythmic ISFs are discussed separately, although in EEG recordings in the ISF frequency band such separation could not be absolute. For example, Figure 2 illustrates three fragments of EEG-ISF recorded at three different electrode locations in a healthy 40-year-old subject during a continuous performance task. Note, that the C3 electrode trace shows almost monochromic oscillations while the infraslow potential at the P3 electrode seems to be driven mostly by irregular sources.

### Rhythmic spontaneous Infra-Slow Fluctuation

We start with reviewing 0.1 Hz oscillations in the scalp recorded EEG. It should be stressed here that 0.1 Hz oscillations are also present in fluctuations of systemic arterial blood pressure (Mayer waves) which in turn are tightly coupled with synchronous fluctuations of efferent sympathetic nervous activity (Julien, 2006). A recent attempt to find a physiological meaning of 0.1 Hz rhythms in EEG was made by simultaneously recording EEG, Near-Infrared Spectroscopy (NIRS), arterial blood pressure, respiration, and Laser Doppler flowmetry (Nikulin et al., 2014). In this study, 0.1 Hz oscillations were detected in 8 out of 10 subjects during rest and showed a striking monochromatic spectrum in the frequency band from 0.07 to 0.14 Hz. The spatial topography of these oscillations was complex, showing multiple foci that were variable across subjects. The authors suggested that these oscillations might be of a rather extra-neuronal origin reflecting cerebral vasomotion (Nikulin et al., 2014).

This suggestion is indirectly supported by observation of a large-amplitude, sinusoidal ∼0.1 Hz hemodynamic oscillations in the cortex of an awake human undergoing surgical resection of a brain tumor by intraoperative multispectral optical intrinsic signal imaging (Rayshubskiy et al., 2014). These hemodynamic ISFs were spatially localized to distinct regions of the cortex, exhibited wave-like propagation, and involved oscillations in the diameter of specific pial arterioles, indicating that the effect was not the result of systemic blood pressure oscillations. Moreover, fMRI data collected from the same subject 4 days before surgery demonstrates that ∼0.1 Hz oscillations in the BOLD signal could be detected around the same region (Rayshubskiy et al., 2014).

A different view suggests that EEG-ISFs are quasiperiodic in nature with different district oscillations emerge in different time periods so that the averaged power spectrum does not exhibit salient peaks (Palva and Palva, 2018).

### Event-related slow cortical potentials

**FIGURE 3:**
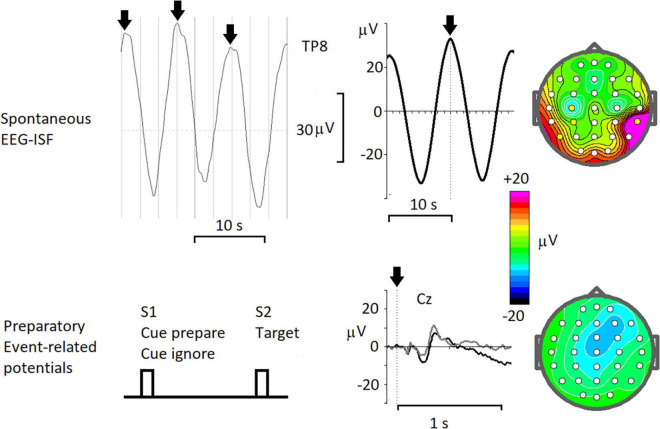
Resting-state spontaneous 0.1 Hz oscillations and task-related slow shifts of the cortically recorded potentials. Spontaneous EEG-ISF- from left to right: a fragment of the spontaneous EEG (< 0.5 Hz) at TP8 electrode in a healthy subject, arrows mark peaks of 0.1 Hz oscillations; averaged oscillations with time 0 corresponding to peaks in spontaneous EEG; map of the averaged potential at 0 time. Preparatory Event-related potentials – from left to right: the scheme of the cued task; the ERPs recorded in a healthy subject for Cue prepare, associated with preparation to the following target (black line) and for Cues ignore, associated with ignoring the trial (gray line); map of the averaged ERP for Cue prepare taken just before the target.

Slow cortical potentials (SCPs) were observed in event-related potentials reflecting preparatory activities of proactive cognitive control. These preparatory activities can last for several seconds and are associated with working memory in general and with sustained attention and motor preparatory sets in particular. These preparatory operations are reflected in CNV (Walter et al., 1964) and stimulus-preceding negativity (for review see Brunia and van Boxtel, 2001). The amplitude of these waves (depending on a particular paradigm and reference electrode) is about 10 μV, which is several times smaller than that of rhythmic spontaneous ISFs (Figure 3). From animal experiments and studies on patients with implanted electrodes (Kropotov and Etlinger, 1999) those preparatory activities are linked to sustained changes of impulse activity of neurons in the corresponding areas. The spatial-temporal differences between rhythmic EEG-ISFs and the slow preparatory negativities indicates that the genesis of these phenomena could be different.

### Arrhythmic infra-slow fluctuations and blood oxygen level–dependent signals

To define the irregular source of EEG-ISFs several studies tried to correlate these electrical sporadic fluctuations with BOLD signals recorded by fMRI (Hiltunen et al., 2014; Thompson et al., 2014; Grooms et al., 2017). For example, both increases and decreases in BOLD signal were shown to reliably follow (with 3 s delay corresponding to the hemodynamic response) increases and decreases in the gamma power (40–100 Hz) of local filed potentials simultaneously recorded in early visual cortices of anesthetized monkeys (Magri et al., 2012).

A first direct observation of relationship between the BOLD signal and the local field potentials was done in animal experiments in 2013 (Pan et al., 2013). In this study, the spontaneous BOLD signal at the recording sites exhibited a significant localized correlation with ISFs of local field potentials as well as with the slow power modulations of higher-frequency oscillations (1–100 Hz) at a 4 s delay compared to the hemodynamic response time under anesthesia. In the following studies in humans, the ISF-EEG signals were shown to correlate with the BOLD signal in spatial patterns that resembled resting state networks while the correlation between ISF-EEG and the BOLD signal varied substantially over time, even within individual subjects (Grooms et al., 2017). A causal relationship between infraslow local field potentials, local oxygen level (pO_2_), and multi-unit activity was recently demonstrated in awake, resting monkeys (Li et al., 2022). In particular, it was shown that changes of pO_2_ and ISF of the local potential in response to neuronal spikes revealed a delayed peak (with a latency of around 6 s) similar to a task-driven BOLD response with oxygen following potential by 0.5 s (Li et al., 2022).

It was demonstrated that independent components of EEG-ISFs were selectively correlated with subsets of cortical BOLD signals in specific fMRI-defined resting-state networks (Hiltunen et al., 2014) and it was consequently speculated that EEG-ISFs are directly associated with the endogenous fluctuations in neuronal activity levels and provide a non-invasive, high-temporal resolution window into the dynamics of intrinsic connectivity networks.

### Arrhythmic EEG-ISFs as correlates of arousal

Performance is modulated by arousal and decreases when arousal levels are too low or too high (Yerkes and Dodson, 1908). Changes in arousal levels are reflected in the galvanic skin response (GSR), pupil size and heart rate (Wang et al., 2018). In a recent study (Sihn and Kim, 2022a) EEG-ISFs and GSR signals were measured simultaneously in human subjects and were shown to have log-log linearity without clear peaks while ISFs phase was coupled with the amplitude of GSR signal. This observation seems to complement the results of a study (Kroupi et al., 2013), where coupling between the phase of the GSR and the amplitude of higher-frequency EEG activity (> 1 Hz) was demonstrated. Simultaneous recording of fMRI and GSR during rest in human subjects observed that the spontaneous fluctuations of BOLD signals are associated with changes in non-specific skin conductance respons (Fan et al., 2012).

### Phases in EEG-ISF

It should be stressed here, that infra-slow fluctuations were also found in behavioral parameters (such as omission errors) in continuous performance tasks (Shalev et al., 2019). Some studies also observed fluctuations of the sustained attention in the theta frequency band (Helfrich et al., 2018). One source of these ISFs might be intrinsic fluctuations in the brain activity (Esterman and Rothlein, 2019).

**FIGURE 4:**
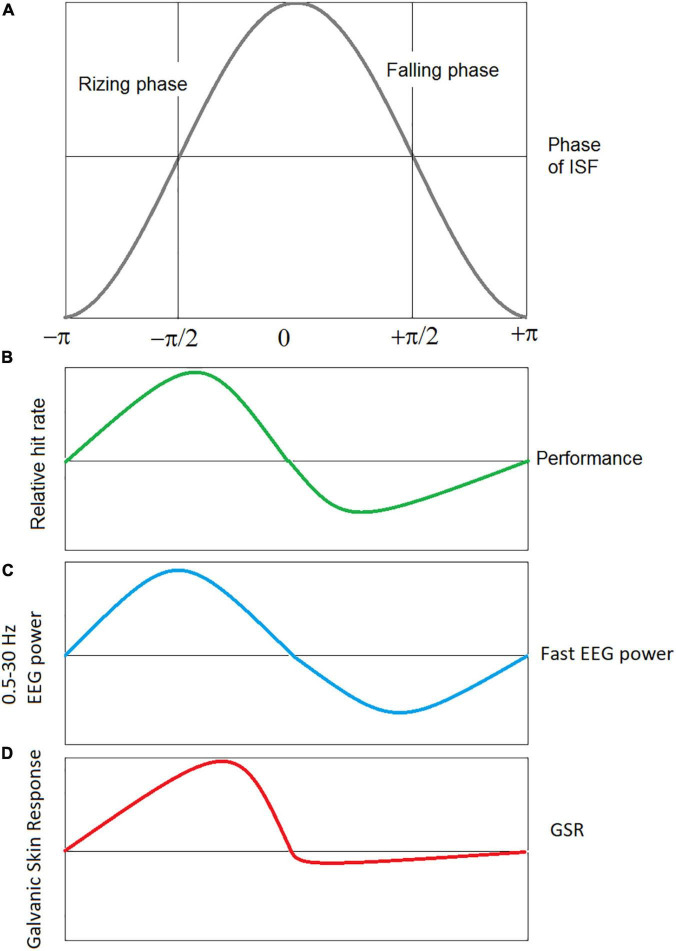
Schematic presentation of correlation of the phases of EEG ISF with performance, power of conventional EEG bands, and GSR signals. **(A)** Infra-slow EEG oscillation with rising and falling phases. **(B)** Performance: hits and missies of near threshold electrical stimulation of the index finger were measured in healthy subjects while recording the full band EEG at Cz (Monto et al., 2008), **(C)** the EEG power in conventional EEG bands (from delta to gamma) is modulated with simultaneously measured ISF-EEG (Monto et al., 2008), **(D)** the EEG-ISF phase correlates with the galvanic skin response (GSR) amplitude, a measure of the arousal level (Sihn and Kim, 2022a).

Simultaneous recordings of behavioral parameters in a continuous performance task and EEG-ISFs demonstrated a specific pattern of relationship of behavioral parameters with the rising and falling phases of EEG-ISFs (Monto et al., 2008; Figure 4). According to some authors, the existence of a functionally meaningful phase indicates the quasiperiodic oscillations in the EEG-ISFs (Palva and Palva, 2012, 2018). Similar, the EEG power in conventional EEG bands (such as delta and gamma) was shown to be differentially modulated by phases of EEG-ISF (Monto et al., 2008). This observation suggests a model that infra-slow processes can be viewed as a broad summation of fast, local neural activity (Breakspear, 2017). However, in contrast, a recent whole-cortex calcium/hemoglobin imaging in mice showed that EEG-ISF is a separate neurophysiological process that is distinct from delta (1–4 Hz) and that is reflected in BOLD signals (Mitra et al., 2018).

EEG-ISFs can be viewed as a measure of cortical excitability. This inference was supported by a transcranial magnetic stimulation (TMS) study demonstrating that the amplitude of motor evoked potentials (MEP) induced by pulses of TMS were sensitive to the phases of EEG-ISF (de Goede and van Putten, 2019). In a recent study (Qiao et al., 2022) an anodal transcranial Direct Current Stimulation (tDCS) on the left dorsolateral prefrontal cortex in the infraslow frequency band (0.05 Hz) enhanced sustained attention more strongly than the conventional tDCS suggesting that this effect was achieved by infraslow modulation of neuronal excitability.

### Traveling infra-slow fluctuations waves

Traveling waves in the infra-slow band were found in hemodynamic, fMRI, and full-band EEG studies. For example, in fMRI studies in humans, ongoing arousal fluctuations were associated with global waves of activity that slowly propagate in parallel throughout the neocortex, thalamus, striatum, and cerebellum (Raut et al., 2021). The authors argue that these traveling waves can explain many features of spontaneous fMRI signal fluctuations, including shaping topographically organized FC networks. Similarly, cortex-wide propagation of neural activity was observed with electrocorticography in macaques (Raut et al., 2021). Traveling waves were also found in hemodynamic measures (Rayshubskiy et al., 2014). These findings suggest that traveling waves spatiotemporally shape brain-wide excitability with global arousal fluctuations indexed by heart rate variability and pupil size (Raut et al., 2021).

### Toward a consistent theory of EEG-ISFs: Testing working hypotheses

#### Avoiding artifacts

Although EEG-ISFs has been known since the 1950s, the question about their physiological basis remains unanswered. This is explained in particular by the fact that the registration of the ISF is a very demanding procedure. The technique includes application of special electrodes, electroconductive gels, amplifiers, corresponding band filters, etc. (Bauer et al., 1989; Tallgren et al., 2005). To avoid skin-related infraslow phenomena (such as sweet artifacts and GSR) the recording of EEG-ISFs requires abrasion (shortcutting) the skin under the electrode (Picton and Hillyard, 1972). Some recent studies, especially during the clinical neurofeedback in the infralow frequency (ILF-neurofeedback) band are not strict regarding these requirements so that rule out the skin-related artifacts in such reports are rather difficult. Inconsistencies in the obtained results regarding the temporal-spatial organization EEG-ISFs might be also due to methodological difference of ISFs analysis, such as the choice of references and parameters of temporal filtration, the functional state of the participants, etc.

Below we formulate the main hypotheses that are supposed to be tested in order to solve the enigma of EEG-ISFs.

#### Decomposing into separate components

In this respect, a working hypothesis can be formulated as follows: EEG-ISFs include several types of activities such as arrhythmic, quasiperiodic, and periodic signals with different functional meanings and potential different mechanisms. The arrhythmic, irregular fluctuations reflect critical-like, scale-free dynamics and are approximated by a power-law function (He et al., 2010; Palva and Palva, 2018). The rhythmic, periodic activities are concentrated around certain frequencies such as 0.1 Hz (Girton et al., 1973; Trimmel et al., 1990; Nikulin et al., 2014) and 0.02 Hz (Watson, 2018). For example, 0.1 Hz rhythms seems to be generated by mechanisms different from that of the arhythmic ones (Nikulin et al., 2014), their local topography indicate that they can’t be directly related to systemic (Mayer waves) and might be associated with local vasomotor processes (Rayshubskiy et al., 2014). To prove or disprove this working hypothesis, a computational procedure to separate the various types of ISFs becomes critical. An application of any blind source separation method such as independent component analysis for such purposes seems to be required (Makeig et al., 1997).

#### Assuming complexity of generators

**FIGURE 5:**
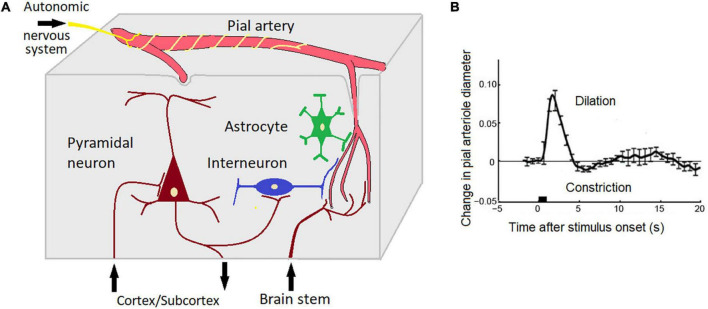
Potential brain sources of EEG-ISF (see text for explanation). **(A)** A scheme depicting interactions between neuronal, glial, and vascular elements. Note that skin-related phenomena such as GSR and skin blood blow are not taken into account; **(B)** The effect of neuronal activity on the diameter of pial vessels as measured with two-photon laser scanning microscopy using a head-fixed rat. The stimulus is a 500 ms puff to the vibrissae. Adapted from Uhlirova et al. (2016). Note that a short sensory stimulation induces an oscillation (deletion, constriction) with a period of 10 s (0.1 Hz).

This hypothesis assumes that several neuronal and non neuronal sources could be implicated in the genesis of EEG-ISFs. Neural sources include changes in the neural membrane potentials caused by activation of cortico-subcortical inputes. The integrated electric current of the corresponding post-synaptic events can generate a portion of the EEG-ISFs. The non-neuronal sources include physiological processes in arterioles and capillary systems in the cortical tissue (Drew et al., 2020) and the electric potentials of the blood-brain barrier (Figure 5). For example, simple hyperventilation may induce large DC shifts with amplitudes up to 2 mV and duration up to several minutes (Voipio et al., 2003). These responses cannot be explained based on any neuronal or glial generator mechanism and, instead, are suggested to be generated across the blood-brain barrier (Voipio et al., 2003). Spontaneous fluctuations in arteriole diameter occur in the resting state within a frequency band centered around 0.1 Hz in mammalian and human brains (Rayshubskiy et al., 2014; Nikulin et al., 2014; Noordmans et al., 2018; Drew et al., 2020). This oscillations in turn drive oscillations in the velocity of red blood cells in the microvessels and oscillations in the partial pressure of oxygen in brain tissue (Mateo et al., 2017). In our studies of patients with implanted electrodes, we observed a strong relationship between infraslow oscillations of local field potentials and local pO_2_ (Kropotov and Grechin, 1975).

#### Applying appropriate functional tasks

To study the functional role of traditional EEG rhythms (alpha, theta…) different functional tasks have been designed. For example, the occipital alpha rhythm was turned out to be sensitive to closing/opening eyes, the mu-rhythms were shown to respond to hand and arm movements, the frontal midline theta was found to be sensitive to the cognitive task load (for a recent review see Kropotov, 2016). As far as EEG-ISFs concern, it’s not clear what are the tasks that modulate those fluctuations.

**FIGURE 6:**
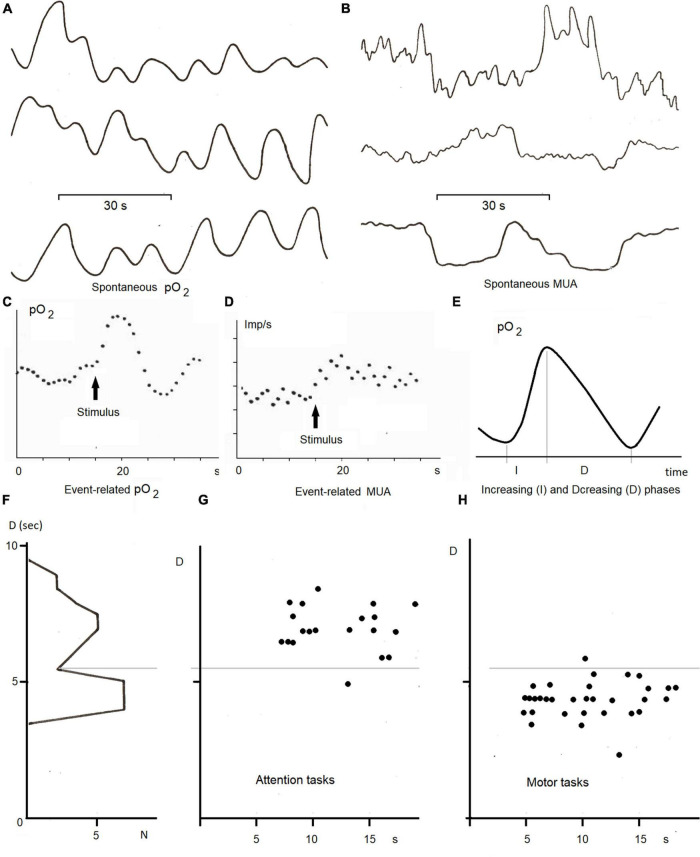
ISFs of local pO_2_ and multiunit activty (MUA) in the basal ganglia and thalamus in the neurological patients with gold electrodes implanted for diagnosis and therapy (adapted from Kropotov and Grechin, 1975; Gretchin and Kropotov, 1979; Kropotov, 1979). **(A)** Spontaneous infraslow oscillations (0.02—-0.1 Hz) of local pO_2_ measured by the polarographic method from different electrodes; **(B)** spontaneous multi-unit activity recorded from different electrodes; **(C)** an averaged event-related pO_2_ in the ventral thalamus in response to ten trials of the arithmetic task, **(D)** a post-stimulu time histogram of MUA in the same task, **(E)** a method of analysis of infra-slow oscillations by measuring duration of increasing (I) and decreasing (D) phases of local pO_2_, **(F)** histogram of D parameter of pO_2_ oscillations in the ventral thalamus and its dynamics during 10 trials of the attention **(G)** and motor **(H)** tasks.

In our studies of ISFs in the concentration of the local pO_2_ recorded from gold electrodes by a polarographic method we used tasks with trials lasting from 30 s up to 90 s (Gretchin and Kropotov, 1979). In these studies the gold electrodes were implanted for diagnosis and therapy in the basal ganglia and thalamic nuclei of Parkinsinian patients. In a motor task the patients were required to flex and unflex an arm, in a memorization task the patients had to memorize a set of 3–4 digits during 60 s and to recall them after 20 s of an interference task. In our studies, the level of local oxygen oscillated in a 0.02–0.1 Hz frequency band (Figure 6A) whereas the increasing and diminishing phases of these oscillations played different functions. The increasing phase took place in response to activation changes in multi-unit activity (Figures 6C,D). In contrast, the decreasing phase of pO_2_ was found to be sensitive to the task performed by the subject (Figures 6G,H).

We presume that a similar methodology could be used in the future studies with a working hypothesis that different parameters of the EEG-ISFs (an increasing and decreasing phase, amplitude of scale-free fluctuations) differently respond to different functional tasks. Another possibility is to use continuous performance tasks with short (around 1–2 s) trials. The consecutive trials in such tasks are not pure independent but weekly auto-correlate so that similar behavioral parameters appeared in clusters (see for example Monto et al., 2008). The estimated frequency of the auto-correlation histograms for hit rates, reaction times and other parameters lies between 0.01 and 0.1 Hz.

#### Searching for neuromarkers of psychiatric disorders

A recent search in the PubMed database showed few studies that used EEG-ISF as neuromarkers for identifying the seizure onset zone (Lundstrom et al., 2021) and for discriminating between the preictal and interictal states in epilepsy (Hashimoto et al., 2021). However, no paper has been found that would use EEG-ISFs as neuromarkers for the discrimination of psychiatric conditions such as ADHD, schizophrenia of those in healthy controls. In view of the increasing use of infralow frequency (ILF) neurofeedback for modulation of psychiatric conditions, future studies must fill this gap.

## Data availability statement

The original contributions presented in the study are included in the article/supplementary material, further inquiries can be directed to the corresponding author.

## Ethics statement

The studies involving human participants were reviewed and approved by the N.P. Bechtereva Institute of the Human Brain of Russian Academy of Sciences, Saint Petersburg, Russia. The patients/participants provided their written informed consent to participate in this study.

## Author contributions

The author confirms being the sole contributor of this work and has approved it for publication.
